# Supplementary material for: Implementing High-Flow Nasal Oxygen Therapy in Medical Wards: A Scoping Review to Understand Hospital Protocols and Procedures
Source: Int J Environ Res Public Health. 2024 May 30;21(6):705. doi: 10.3390/ijerph21060705 (PMC11203406; doi:10.3390/ijerph21060705)
Supplement: Supplementary file 1 [file ijerph-21-00705-s001.zip › ijerph-2988962-supplementary.pdf]

Supplementary Material

Table S1. Further characteristics of included LHGD.

	Count (n)	Frequency (%)
Document contributors *		
-Not specified	9	34.6
-Nursing staff	12	46.2
-Medical staff	7	26.9
-Physiotherapy staff	2	7.7
-Other	11	42.3
Year of LHGD publication		
Not specified	4	15.4
2021–2022	3	11.5
2019–2020	4	15.4
2018 or prior	15	57.7
Date of last review		
Not specified	8	30.7
2023–2024	7	26.9
2019–2022	9	34.6
2018 or prior	2	7.7
Document length (pages)		
0–5	6	23.1
6–10	8	30.8
11–15	9	34.6
16–20	3	11.5
LHGD objective stated	23	88.5
Definitions provided	16	61.5
LHGD summary provided	16	61.5
Nasal high flow system components specified	21	80.8
Guidance for choice of nasal prong size	11	42.3
Advantages of nasal high flow provided	22	84.6
Guidance for patient positioning provided	15	57.7
Nurse to patient ratio while on nasal high flow		
Not specified	24	92.3
Clinical discretion	2	7.7
Maximum recommended FiO_2 on general ward		
Not specified	9	34.6
40–49%	3	11.5
50–52%	14	53.8
Maximum recommended HFNO flow on general wards		
-Not specified	7	26.9
30–39 LPM	12	46.2
40–49 LPM	4	15.4
50–60 LPM	3	11.5
Flow targets before cessation of HFNO for patients with COPD		
Not specified	16	61.5
20 LPM	8	30.8
25 LPM	2	7.7
Oxygen source after weaning HFNO		
Not specified	19	73.1

Conventional oxygen therapy	4	15.4
Either conventional oxygen therapy or room air	3	11.5

* Categories are not mutually exclusive and cumulative percentages may sum to over 100. SpO_2: Pulse oximetry oxygen saturation, HFNO: High flow nasal oxygen LPM: Litres per minute, FiO_2: Fraction of inspired oxygen, COPD: Chronic Obstructive Pulmonary Disease.
